# The Giant of Attenuation

**Published:** 1892-09

**Authors:** Hayes C. French

**Affiliations:** San Francisco, Cal.


### THE GIANT OF ATTENUATION.

Hayes C. French, M. D., San Francisco, Cal.

When we remember the diversity of opinion in our ranks upon the subject of attenuations and the extreme and rapid changes of practice in regard to potencies by the same individual, the reason for the wide variation becomes a question of deep interest to every philosophical mind. No doubt in many the adherence to a high or low potency is the result of profound conviction in favor of their choice and opposition to any other from the beginning of practice, and when we consider the trivial accidents by which the convictions and prejudices of a lifetime have been overcome and stalwart enemies been converted into loyal devotees to Homœopathy, it seems certain that the status of individual opinion in reference to potency may often be a matter of environment or education. Unquestionably many relapses from the finer to a cruder use of our drugs are due to the growth and push of business, necessitating a rapidity of prescription which is incompatible with the proper selection of a remedy. Every true follower of Hahnemann has to confront a constant tide of ridicule against high potencies, both from our own ranks (which is the most unkind), and from the ranks of our acknowledged enemy, which has a tendency to weaken loyalty in all but the most stalwart. It is our belief that the fate of Homœopathy will depend upon our falsity or fealty to the single and attenuated remedy, and observation will convince any thoughtful mind that it is only a step from the habitual and exclusive use of colored tinctures to polypharmacy, and only another to the practice of more liberal allopathy. We are forced to the shameful admission that the prescription-files of many allopathic drug stores point to the fact that with many of its professed adherents Homœopathy is only a *name*, and some are even ashamed of *that*. The noblest patriot and statesman of any age, at a crucial period in our national history, made the prophetic utterance that a house divided against itself could not stand, and that this Union could not continue to exist half slave and half free, but that ultimately it must become all one thing or all the other. Your writer claims none of the attributes of a prophet, but firmly believes that the advocates of Homœopathy must stand more steadfastly on the demonstrated truth of its distinctive law of cure or it will lose its power and all semblance of cohesion, and gradually but inevitably merge into the dominant school, and we could desire, in regard to the future of Homœopathy, the sublime faith of the immortal Lincoln when he said, "*I do not expect the house to fall.*"

While the writer would not exclude himself from many of the articles of this arraignment, he can truthfully say that never since his abandonment of the old-school therapeutics has he failed to find his most brilliant successes in the use of the higher potencies. We have a drawer of what were originally sixth and twelfth decimal potencies, and another of the thirtieth centesimal attenuation, which for the past thirteen years have been stretching out into the potent ether of infinitesimalism, and yet in spite of our confessed crudity in emergencies, and especially in tough chronic cases that have been the rounds, we return to these indefinitely attenuated remedies, and our two hundred thousandths which have been similarly treated, after a careful study of the materia medica with a confidence that is constantly gaining strength from demonstration. If the symptoms in any case point unequivocally to a certain drug, and a low potency fails of results, we would consider it little less than criminal to change the remedy before going higher. In cephalalgia, in obscure eye troubles in which the mental and subjective symptoms predominate, and in which general and indiscriminate drugging has been employed, we should have no confidence in any but the single remedy, and a relatively high potency. In malarial troubles that have been suppressed by Quinine, or rendered insufferable by fruitless guessing through the lower powers of homœopathic remedies, we would turn to high potencies with an assurance born of oft-repeated triumphs.

Case I.—A family whom we had won to Homœopathy by successful treatment in this city, moved to Marysville, and after a two years' residence in the society of malarial microbes, the mother, who had been a teacher, after giving a most dismal picture of the demon of ague which possessed the whole family and had repeatedly returned with renewed vigor after suppression with large doses of Quinine, in desperation, and with many doubts, wrote to ask if homœopathic remedies had any such influence over ague as she had seen manifested in eye cases. The whole family were suffering from the same general type of tertian ague: chill in the early forenoon with intense thirst, often followed by nausea; yawning, stretching, and aching in the bones, though varying in degree; no heat during the chill, and little or no sweat. Four powders of Eupatorium-perfoliatum$^{200}$ were sent, with orders to dose the whole family at intervals of four hours, from the same glass. Some weeks later the gratifying report came that the first powder had cured the entire family, and the three remaining ones were being treasured as charms against any future invasion, and in no case did the fever recur.

Case II.—A boy of three years was sent from the Sacramento Valley suffering from typho-malarial fever and headaches supposed to be in some way associated with retinal hyperæsthesia on account of the great photophobia. He had enjoyed the regulation diet of Quinine. After dosing him for several days with low potencies, and no results, one dose of Sulphur$^{81M}$ was given as a John-the-Baptist, and a most careful and exhaustive study of the symptoms was made. The case was somewhat obscure and ill-defined, but there was a tendency to quotidian recurrence at about ten A. M., with insatiate thirst, and hydroa on the lips had given place to dry crusts. Chill not marked, lasted till about noon, with weakness and prostration, and followed by fever and dry, hot skin till evening, with moderate sweat lasting through the night. The child was emaciated and sallow, with spleenic enlargement and induration. Apyrexia not well marked. Nat-mur.$^{200}$ was given every three hours till four doses were taken, followed by placebo, and the fever was cured, with no recurrence.

Case III.—Maiden lady of about thirty-eight, motive temperament, dark hair, and suffering from brain fag and general exhaustion. She had lived in a malarial country and taken Quinine ineffectually for a typho-malarial fever. Nat.-mur.$^{200}$ modified the case, stopping the chills and nightly sweat, and Rhus-tox.$^{200}$ completed the cure. The red tongue with the characteristic restlessness of Rhus led to that remedy, and the cure was rapid and complete.

